# Epidermal Stem Cells and Their Epigenetic Regulation

**DOI:** 10.3390/ijms140917861

**Published:** 2013-08-30

**Authors:** Qi Shen, Hongchuan Jin, Xian Wang

**Affiliations:** Department of Medical Oncology, Institute of Clinical Science, Sir Runrun Shaw Hospital, School of Medicine, Zhejiang University, Hangzhou 310058, China; E-Mails: brilliasq@163.com (Q.S.); jinhc@zju.edu.cn (H.J.)

**Keywords:** epidermal stem cell, epigenetic modification, histone methylation, DNA methylation, noncoding RNA, microRNA, long noncoding RNA

## Abstract

Stem cells play an essential role in embryonic development, cell differentiation and tissue regeneration. Tissue homeostasis in adults is maintained by adult stem cells resident in the niches of different tissues. As one kind of adult stem cell, epidermal stem cells have the potential to generate diversified types of progeny cells in the skin. Although its biology is still largely unclarified, epidermal stem cells are widely used in stem cell research and regenerative medicine given its easy accessibility and pluripotency. Despite the same genome, cells within an organism have different fates due to the epigenetic regulation of gene expression. In this review, we will briefly discuss the current understanding of epigenetic modulation in epidermal stem cells.

## 1. Introduction of Epidermal Stem Cells

The function of stem cells is appreciated to be crucial throughout life. For instance, cell loss is continuous in the epidermis, through the sloughing off of pavement epitheliums, the secretion of sebum, and the cycle of hair. Therefore, it is necessary for a population of stem cells residing in the epidermis to proliferate, differentiate and replace those lost cells. Epidermal stem cells, by definition, reside in epidermal niches and replenish the cells eliminated in the skin [1]. Other stem cells play a similar role to maintain regeneration in other tissues, however, due to their superficial location, epidermal stem cells are more easily accessible and thus provide a highly valuable tool for either stem cell research or regenerative medicine.

Basically, stem cells contain induced pluripotent stem cells, embryonic stem cells, and adult stem cells. Induced pluripotent stem cells are a type of pluripotent stem cell artificially induced from a non-pluripotent cell such as adult somatic cell by “forced” expression of specific genes. Such artificial stem cells offer an endless source for stem cell research as well as organ transplantation. Embryonic stem cells, isolated from the inner cell mass of an early-stage embryo blastocyst, are distinguished by two distinctive properties, the pluripotency and the ability to replicate indefinitely. The term pluripotency refers to the fact that embryonic stem cells are capable of differentiating into all derivatives of the three primary germ layers, ectoderm, endoderm, and mesoderm [2]. Each germ layer contains more than two hundred cell types in the adult body. Embryonic stem cells have been used in research for many years and employed for an impressive number of diseases including: immune-system related genetic diseases [3], neurodegenerative disorders [4], diabetes [5], spinal cord injuries [6] and so on. It seems that there is a brilliant and bright future for applications of embryonic stem cells. However, the ethical concerns of embryonic stem cell use have continued for several years. In addition, cancer-inducing risks [7] and graft-*versus*-host diseases associated with allogeneic stem cell transplantation [8] involved in the embryonic stem cell therapy further hinder the wide application of embryonic stem cells. Therefore, adult stem cells have emerged as the alternative of embryonic stem cells. Unlike the pluripotency of embryonic stem cells, the multipotency of adult stem cells depicts a differentiating capacity of stem cells restricted to several limited categories of daughter cells instead of all types of progeny cells in the adult body. The main challenge for adult stem cell based therapy is the harvest and propagation of adult stem cells. As a member of the adult stem cell family, epidermal stem cells have the potential to generate diversified types of epidermal cells. Distinct from the plasticity and pluripotency of the embryonic stem cells, each epidermal stem cell population located in each niche only feed a restricted differentiated lineage for the renewal of the different components of epidermis [9]. This characteristic permits epidermal stem cells prevalently used for skin grafting, in particular following large burns [10].

### 1.1. Identification of Epidermal Stem Cells

Classical approaches to identify epidermal stem cells *in vivo* take full advantage of their slow-cycling character, the most conspicuous features besides their high proliferative potential. One approach to identify epidermal stem cells is to label DNA or chromosomes in all dividing epidermal cells and then to track the ones that do not divide afterward with the label retaining until adulthood [11]. In early studies, incorporation of artificial material of DNA synthesis such as 5-bromo-2′-deoxyuridine (BrdU) which can be detected by immunofluorescence with anti-BrdU antibodies or 3H-thymidine (3HTdR) which requires long-time of radiation exposure provided tracking labels [12,13]. As histones are the principal structural proteins of eukaryotic chromosomes, the H2B-GFP (green fluorescent protein) fusion protein incorporated into nucleosomes is used for fluorescent chromosome labeling [14] to mark infrequently cycling stem cells. Transgenic mice which express H2B-GFP under control of a tetracycline-responsive regulatory element (TRE) are engineered to track the fate of label retaining cells, by tracing H2B-GFP fluorescence intensities relative to the proliferation-associated markers Ki67, phosphorylated histone H3 (p-H3) and basonuclin (BSN). Semiquantitative fluorescence proves that GFP-high and Ki67-, p-H3-, and BSN-low cells correspond in fluorescence intensity to bulge cell location, whereas GFP-low fluorescence cells place themselves outside the bulge [15]. This coincides with the hypothesis that bulge is a growth and differentiation—restricted epidermal stem cells niche. Although no method can ensure that all stem cells are labeled owing to the possibility that a stem cell did not synthesize DNA during the labeling period and thus will never be regarded as a LRC [16], label retention based methods play important roles in the identification of epidermal stem cells, and the confirmation of their location *in vivo*.

Another kind of identification approach is the clonal analysis after cell culture *in vitro*. When transplanted to the dermo-epidermal junction of newborn mouse skin, a single epidermal stem cell would produce a whole or part of developing hair follicles (HF) after more than one hundred cell divisions [17]. This provides convincing evidence that special cell culture after single cell isolation could identify epidermal stem cell. Together with a burst of studies on epidermal stem cells, novel identification methods, such as label-free detection strategies based on nucleic acid aptamers are being developed continuously [18].

### 1.2. Functional Characteristics of Epidermal Stem Cells

As mentioned above, slow cycling or rarely cycling and high proliferation are the most distinctive characteristics of epidermal stem cells. In addition, epidermal stem cells are ultrastructurally primitive and lack the expression of any differentiation-related keratins or other markers. Furthermore, a cluster of epidermal stem cells affiliated with a specialized mesenchyme reside in specialized stromal niches to protect themselves from physical destruction and melanin stimulation [19]. To maintain the homeostasis of skin, cells lost during the course of turnover are replenished by cells generated in the basal layer. Two seemingly contradictory hypotheses have been put forward to explain how the equilibrium is established. On the one side, adult epidermal homeostasis is based on the basal layer including at least two distinct subpopulations of cells, the epidermal stem cells and the surrounding transit amplifying (TA) cells. These two proliferative subpopulations differ in the length of cycle since the cycle of the stem cells is much longer [20]. The epidermal stem cell supports clonal units of transit amplifying (TA) and differentiated cells which organize in the so-called epidermal proliferative units (EPU). An EPU embodies approximately ten basal cells sustained by a single self-renewing stem cell. However, progenitor cells which are capable of generating both hair follicles and interfollicular epidermis located in the hair-follicle bulge appear of no use in maintaining normal interfollicular epidermis. Besides, this model indicates that the basal—layer clone—size distribution should be dependent of time and emerge as a single epidermal proliferative unit. This hints at a sharp contradiction with the progressive increase in average clone size observed in the epidermis. The other side insists on that only one type of progenitor cells undergoes both symmetric and asymmetric division at certain ratio to guarantee epidermal homeostasis [21]. After division, one of the daughter cells of the stem cell remains to be a stem cell while the other differentiates without any further rounds of cell division. Recent studies indicate the hierarchical organization and the proliferation dynamics of two distinguishing classes of predecessor cells that play different roles to maintain homeostasis in adult mice epidermis. Moreover, both the slow-cycling stem cells and the more rapidly cycling committed progenitor cells share a similar pattern of asymmetric self-renewal. In other words, cells reach a balance between proliferation and differentiation through random fate determination. These findings provide a reconciliation of two seemingly contradictory theories of epidermis maintenance [22]. Proponents propose that it is a reasonable way to explain the proliferative heterogeneity previously reported in epidermis—slow-cycling epidermal stem cells can switch rapidly and reversibly between quiescence and activation following injury or drug treatments [23]. This partitioning of function, progenitors to undertake routine homeostatic renewal and quiescent stem cells in response to various damages, may represent a universal strategy of tissue maintenance not only in epidermis but also in other tissues, such as cornea [24], blood [25], muscle [26] and brain [27].

### 1.3. Location and Classification of Epidermal Stem Cells

**Figure 1:**
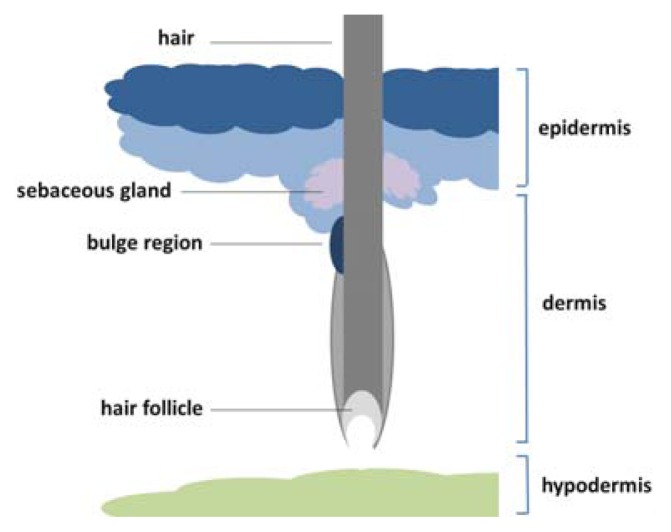
A cross section of the human skin. Epidermal stem cells are found in the basal layer and in the bulge region.

The skin epidermis protects mammals against a number of environmental stresses, such as water loss and microorganism infection. The skin is constituted of three primary layers, the epidermis, the dermis and the hypodermis (Figure 1) [28]. The epidermis has the ability to elaborate the body surface with appendages ranging from hair follicles and nails to different glands such as sebaceous glands [29]. The basal layer of epidermal cells proliferates and differentiates upward till the outmost layer stratum corneum layer to shape the stratified squamous epithelium. Cells leave the basal layer and move towards the skin surface through terminal differentiation [30]. Epidermal stem cells most likely reside within the basal layer of the stratified epithelium close to basement membrane which is rich in growth factors to maintain the stemness of epidermal stem cells [31]. Epidermal stem cells come out from the niche and move to the outmost layer by forming intermediate product of spinous cells to the end outermost product, dead enucleated cells outermost [32]. It has been estimated that a cell coming from the basal layer to the cornified layer will take the minimum time at around one to two weeks. The term niche refers to a reservoir region that can protect the stem cell population from the environmental damage and pigmentation. The niches show no distinct morphology in the epidermis. However, a burgeoning viewpoint of epidermal stem cells is that there appears to be at least three distinguishing niches for epidermal stem cells, the follicle bulge, the base of the sebaceous gland and the basal layer of the epidermis [33], among which follicle bulge is the most well-studied one. Hair follicles could replenish multiple cell lineages at the commencement of each new anagen cycle, suggesting the presence of a reservoir of stem cells in the hair follicle contains [32]. Approximately 150 genes preferentially expressed in the bulge relative to the proliferating basal cells of the epidermis. Among these bulge-specific genes, transcription factors such as Nfatc1 [34] and Lgr5 [35] are up-regulated. However, different stem or progenitor cells in different niches may have different gene expression patterns. For example, transcription repressor Blimp-1 (B lymphocyte maturation-induced protein-1) is expressed in progenitor cells in the base of the sebaceous gland [36].

### 1.4. Signaling Pathway Implicated in the Epidermal Stem Cells

In recent years, several conserved signaling pathways have been confirmed to be essential for epidermal stem cell homeostasis, differentiation and proliferation. The Notch, Wnt/β-catenin, c-myc and p63 pathways constitute the core network of epidermal stem cell maintenance. Notch signaling pathway is initiated by receptor-ligand interaction between cells and activated by subsequent cleavage of Notch into NICD (Notch intracellular domain) by TNF-α converting enzyme (TACE) and γ-secretase. Consequently, NICD converts the transcription factor CBF1/CSL from a gene repressor to a gene activator, thus initiating the transcription of downstream target genes such as Hes and Hey family. Notch receptors are not expressed in the suprabasal cells of the interfollicular epidermis (IFE) and the hair follicle. The cell cycle regulator p21 is upregulated by Notch1 to initiate terminal differentiation by inducing cell cycle arrest in proliferating keratinocytes. Meanwhile, Notch signaling also regulates cell adhesion and influence cell localization during terminal differentiation of keratinocytes [37]. Once Notch signaling was inhibited, skin cancer was developed probably through the suppression of cell differentiation [38]. In addition, Notch signaling interacts with other signaling pathways like Wnt/β-catenin, c-myc and p63 to regulate skin homeostasis. Wnt signaling is necessary for hair follicle formation and their postnatal maintenance. It could affect the expression of some genes in Notch signaling pathway [39], while deletion of Jagged-1 could blockβ-catenin induced hair follicle formation [40]. Furthermore, through the downregulation of selected interferon-responsive genes, the activation of Notch signaling can inhibit the expression of p63 which is implicated in establishment of the keratinocyte cell fate and/or maintenance of epithelial self-renewal. In turn, p63 counteracts the ability of Notch1 to restrict growth and promote differentiation by functioning as a selective modulator of Notch1-dependent transcription and function [41]. However, a more complex crosstalk between p63 and Notch was discovered in mammary gland. Overexpression of p63 promotes cellular quiescence with an unexpected principle mediator as Notch 3, which is one of four Notch family members expressed in mammals [42]. Therefore, both cellular context and other collaborating signaling pathways likely affect the consequence of p63-Notch crosstalk.

## 2. Epigenetic Regulation of Epidermal Stem Cells

As a response to dynamic environmental conditions, the interaction of numerous signaling pathways will eventually remodel the epigenetic regulatory network to fluctuate gene expression but leave genome physically unchanged. Fifty years ago, Waddington proposed an epigenetic landscape model to describe the cell differentiation from stem cells as the trajectory of a ball into branching valleys [43]. It was updated recently to emphasize the self-renewal and plasticity of stem cells. Temporal oscillations in gene expressions restricted the cellular state to a certain region in a fixed state or a set of dynamically changing states [44]. Cell–cell communication in addition to autocrine or paracrine cytokines was suggested to regulate the fate determination of stem cells through epigenetic regulatory network to control dynamic expression of some particular genes [45]. The epigenetic network mainly consist of three major events, histone modifications especially histone methylation or acetylation, DNA modifications mainly DNA methylation and noncoding RNAs. Development of a probabilistic model to cluster genomic sequences based on the similarity of temporal changes of multiple epigenomic marks reveals a variety of rules of dynamic gene regulation during epidermal stem cell differentiation and proliferation [46].

### 2.1. Histone Modifications and Epidermal Stem Cells

One molecular root of the counterpoise between stemness and differentiation of epidermal stem cells is the interplay of histone modifications with tissue-specific transcription factors and other epigenetic regulators. A group of epigenetic regulators such as Polycomb group (PcG) proteins are required for epigenetic silencing of lineage-defining genes to maintain stemness of epidermal stem cells [47]. There are two main complexes, the Polycomb Repressive Complexes-2 (PRC2) and the Polycomb Repressive Complexes-1 (PRC1). The PRC2 complex is comprised of three subunits, Ezh2/Ezh1, Eed and Suz12. Ezh2 or its paralog Ezh1 modulates tri-methylation of histone H3 at lysine 27 (H3K27me3), which is a chromatin modification related to transcriptional repression [48]. Meanwhile, the PRC1 complex consist of four subunits, Cbx (Cbx2-4-6-7-8), RING1 (RING1A/B), PHC (PHC1-2-3) and PCGF (PCGF1-6) [49]. The antagonistic actions between Polycomb and Trithorax are responsible for appropriate cell fate determination in epidermis while the differentiation-associated transcription factor GRHL3/GET1 recruits the Trithorax complex to several differentiation-associated genes [50]. The synergistic or antagonistic interactions between PRC1/2 and histone modifications like H3K27me3, H3K36me3, H2A.Z, H3K4me1/2/3, H3K9me3 or H3K27ac, are proven essential to preserve stemness and regulate differentiation in epidermal stem cells.

Though covalently modification of histone tails, the PcGs turn to be obstacles of transcriptional activity. After nonenzymatic sticking to the chromatin, Ring1 in PRC1 mono-ubiquitylates histone H2A at lysine 119 [51]. This modification is dispensable for its target binding but indispensable for efficient repression of target genes to maintain stemness of epidermal stem cells [52]. There are two categories of PRC1 complexes been identified. One contains the Cbx subunit and depends on H3K27me3 for chromatin adherence. The other has RYBP and binds to the chromatin independent of PRC2 and H3K27me3, overturning the orthodoxy that recruitment of PRC1 to chromatin depends on its concerted action with PRC2 [53]. Cbx4, another PRC1-associated protein, maintains human epidermal stem cells as slow-cycling and undifferentiated to protect the epidermal stem cells from senescence [54].

The other polycomb complex PRC2 catalyzes trimethylation of H3K27me3, and its subunit Ezh2 prevents the differentiation of basal epidermal cells by precluding the binding of the pro-differentiation AP-1 transcription factor to late differentiation genes [55]. Knockout of Ezh2 in embryonic basal epidermal stem cells totally eliminates the presence of H3K27me3 mark but moderately affect the fate determination of epidermal stem cells. The changes induced by knockout of Ezh2 could be found during embryogenesis and in early postnatal epidermis rather than in epidermis of an adult. Moreover, Ezh2 expression is decreased in adult epidermis. Different epidermal stem cells within one Polycomb-dependent tissue respond differently to loss of H3K27me3. Despite striking phenotypic difference of HF suspended morphogeny and epidermis excessive hypermorphosis, similar genes are up-regulated in HF and epidermal Ezh1/2-null progenitors. The PcG proteins and Ezh2 can be induced by Twist-1, resulting in an increase of H3K27me3 on the Ink4A/Arf locus [56]. The proliferation or survival of Ezh1/2-null HF progenitors was restored after transduction of Ink4b/Ink4a/Arf shRNAs, suggesting the relevance of this locus to the HF phenotypes [57].

Jarid2 relates to all of the known canonical PRC2 components by a conserved physical interaction with PRC2 and has a major effect on PRC2 recruitment. Besides, *in vivo* studies revealed that Jarid2 mutants affected only H3K27me3 but no other histone modifications [58]. However, the details of PRC1 and PRC2 recruited to genes are not fully apprehended. There is an evidence for an interaction of the transcription factor REST with PRC1 and RC2. REST has context-dependent functions for PRC1- and PRC2- recruitment and also function as a limiting factor for PRC2 recruitment at CpG islands [59].

More than the role in the preservation of stemness, PcGs were recently found to be involved in the regulation of cell differentiation. Release from Polycomb repression only partially explains the activation of differentiation genes. Stable knockdown of SUZ12, a cornerstone for PRC2 assembly and function, leads to a significant precocious expression of a subset of terminal differentiation markers in intestinal cell models. This identifies a mechanism whereby PcG proteins participate in slow down terminal differentiation in the TA cell population [60]. Similarly, loss of polycomb-mediated silencing may enable the upregulation of repair-related genes and stimulate the epidermal stem cells to initiate terminal differentiation [61].

Generally, transcriptionally upregulated genes are marked by H3K36me3 in gene bodies, H3K4me3 and H3K9ac on promoters and H3K27ac and H3K4me1 in enhancer regions [62]. Recent studies show that H3K36me3 affiliating to polycomb-like (PCL) proteins PHF19 leads to the recruitment of PRC2 and subsequently de novo gene silencing. Coexistence of H3K36me3, H3K27me3, and PHF19/PCL3 at a subset of poised developmental genes is identified in murine mutipotent stem cells. PHF19/PCL3 Tudor motif is required for the recognition of H3K36me3 to promote the intrusion of PRC2 complexes into active chromatin regions and consequently gene silencing [63]. The combined activities of KDM5a (and possibly KDM5b), plus NO66 and/or KDM2b may remove both marks of transcriptionally active genes, H3K4me3 and H3K36me3, facilitating PcG-mediated silencing of previously active genes [64].

In addition, histone variant H2A.Z plays essential roles in mediating nucleosome depletion and recruiting transcription cofactors to cis-regulatory elements [65]. In an *in vivo* mouse hair follicle stem cell model, H2A.Z shows specific immunodetection on immortal DNA chromosomes, indicating H2A.Z as an asymmetric self-renewal-associated (ASRA) biomarker. Its mRNA is significantly downregulated during asymmetric self-renewal compared to symmetric self-renewal [66]. H2A.Z is highly enriched at promoters or enhancers and is required for both self-renewal and differentiation. In self-renewing stem cells, knockdown of H2A.Z compromises OCT4 binding to its target genes and leads to decreased binding of MLL complex to active genes and of PRC2 complex to repressed genes. H2A.Z can also accumulations at developmentally silenced genes in a polycomb independent manner [67]. In addition, inhibition of H2A.Z also compromises RA-induced RARalpha binding, activation of differentiation markers, and the repression of pluripotency genes during differentiation of stem cells. Therefore, H2A.Z acts as a ying-yang facilitator to regulate the access of both activating and repressive transcriptional factors [68].

In addition to histone methylation, histone acetylation also exerts significant influences in stem cells. Either histone acetyltransferase (HAT) or histone deacetylase (HDAC) dedicates to the network of chromatin modification in epidermal stem cells. MOZ (monocytic leukemia zinc-finger protein) and MORF (MOZ related factor) are catalytic subunits of HAT complexes essential in stem cell developmental programs. The canonical HAT domain of MORF/MOZ follows a tandem of plant homeodomain (PHD) fingers. The tandem PHD fingers of MORF recognize the *N*-terminal tail of histone H3 while acetylation of Lys9 (H3K9ac) or Lys14 (H3K14ac) enhances this binding [69]. During differentiation and survival of epidermal keratinocytes, peroxisome proliferator-activated receptors (PPARs) play a key role as well. p65/RelA represses PPARdelta-dependent transactivation in a HDAC activity dependent manner [70]. EGFR-ERK pathway is also implicated in advanced stages of epidermal differentiation at least in part through modulating HDAC activity [71].

Interacting with chromatin remodeling factors, transcription factors such as p63 and Myc have some modulatory impacts on the differentiation and proliferation of epidermal stem cells. IFE and HF develop from surface ectodermal progenitor cells. Deletions of ectodermal HDAC1 and HDAC2 result in failure of HF specification and epidermal stratification, paralleling with the loss of the important ectodermal transcription factor p63 [72]. P63 makes use of several chromatin-remodeling proteins to exert its function in epidermal embryogenesis and adult epidermal homeostasis. To repress expression of anti-proliferative genes, p63 requires HDAC1/2 and the meCpG stabilizer Lsh/HELLS [73]. The exiting of epidermal stem cells from their niches and terminal differentiation is dependent on activation of Myc and subsequently chromatin modifications. Quiescent stem cells in the IFE and the hair follicle bulge have high levels of H3K9ac or H3K14ac. With the aid of HDAC activities, Myc can initiate the increased acetylation at histone H4 and mono-methylation at lysine 20 which then switches to chromatin silencing epigenetic modifications di-methylation at histone H3 lysine 9 and histone H4 lysine 20 [74]. In addition, an androgen receptor/p53 axis is also involved in c-MYC-induced sebaceous gland differentiation [75].

### 2.2. DNA Modification and Epidermal Stem Cells

Another category of epigenetic modification in epidermal stem cells takes place on the genomic DNA. 5-Methylcytosine (5-mC) is identified as a crucial and prevalent epigenetic DNA modification involved in the development [76]. Genomic methylation patterns in somatic differentiated cells are generally stable and heritable, ensuring tissue-specific gene expression in a heritable manner throughout development. Therefore, methylation of DNA at CpG islands, which is associated with transcriptional repression, is regarded as hereditary imprints [77]. De novo methylation of DNA is performed by DNA (cytosine-5-)-methyltransferase 3a (DNMT3a) and DNMT3b while DNMT1 is responsible for methylation maintenance [78].

While early studies reveal that DNMT1 is dispensable for embryonic stem cell maintenance [79], recent studies focused on whether DNMT1 makes a determinate action for maintaining the progenitor state in the epidermis [80]. Paralleling with a role in the spatial-upward epidermis differentiation process, DNMT1 is mainly confined to cells in the basal layer of adult epidermis where epidermal stem cell located and is lost in outer differentiated layers. DNMT1 knockdown leads to premature differentiation within the progenitor-containing compartments and defects in tissue self-renewal [80]. Genome-wide analysis revealed that a significant portion of epidermal differentiation gene promoters are methylated in self-renewing. All of these evidences point to a fact that DNMT1 is essential for the function of epidermal stem cell, mainly in preservation of progenitor state and trigger of exit from the niche [81]. UHRF, a component of the DNA methylation machinery that targets DNMT1 to hemi-methylated DNA, is also necessary to suppress premature differentiation and sustain proliferation [82]. Lsh, a chromatin remodeling protein, can interact directly with DNMT3a and DNMT3b or indirectly with DNMT1 via DNMT3b [83] and participate in DNA methylation [84]. Mice with a K14-Cre-mediated loss of DNMT1 show an uneven epidermal thickness, alterations in hair follicle size and shorter and thinner hair fibers [85]. These results highlight the significance of DNA methylation in HF regeneration.

As 5-methylcytosine (5-mC) may be further modified by TET family [86] to 5-hydroxy-methylcytosine (5-hmC) in embryonic stem cells [87], it provides a new avenue to look into the epigenetic modulation of stem cells as a potential mechanism leading to active DNA demethylation. Enrichment of 5-hmC at enhancers marked with H3K4me1 and H3K27ac suggests 5-hmC is probable implicated in the regulation of some specific promoters and enhancers related to stem cell function [88]. 5-hmC modification is prevalent in embryonic stem cells and neurons [89], but the distribution of 5-hmC in epidermis has not been rigorously explored. High levels of 5-hmC are reported to be lost during differentiation, but reappear during the generation of induced pluripotent stem cells, suggesting that 5-hmC enrichment associates with a pluripotent cell state [90]. As the neurons and epidermis have some common in the tissue origination and the above-mentioned remarkable dynamic change of 5-mC during the epidermal stem cell exiting from the niches, it is reasonable to make an audacious speculation that if some intriguing changes of 5-hmC would arise in epidermal stem cells process. In differentiated nonreplicating somatic cells, DNA methylation can also be reversible although it is still unknown whether active demethylation is involved [91].

### 2.3. Noncoding RNAs and Epidermal Stem Cells

It is less than ten years when noncoding RNAs including micro-RNAs (miRNA) and long noncoding RNAs (lncRNAs) have been appreciated to regulate epidermal stem cell identity, proliferation and differentiation. In 2006, Yi *et al.* cloned more than 100 miRNAs [92] from skin and showed that epidermis and HF differentially express discrete miRNA families. MiRNAs orchestrate the formation of epidermis and skin appendages. The multifunctional enzyme Dicer is required in the processing of miRNAs and their assembly into the RNA-induced silencing (RISC) complex. Its deletion in embryonic skin progenitors has an impact on developing hair germs to evaginate layers rather than invaginate, thereby perturbing the epidermal organization. In the Dicer mutant, no normal hair shafts are produced, together with the degenerated follicles lacking stem cell markers although the epidermis is hyperproliferative. These results implicate the vital roles for miRNAs in epidermis and HF development [93]. Several miRNAs, particularly *miR-203*, have been confirmed to be crucial to the differentiation and proliferation in epidermis primarily through directly or indirectly targeting epigenetic regulators or transcription factors.

*MiR-203* is the well-studied miRNA in epidermis biology. It is upregulated in cultured keratinocytes after differentiation inducers treatment [94,95]. *MiR-203* promotes epidermal differentiation by restricting proliferative potential and inducing cell-cycle exit. The targets of *miR-203* include suppressor of cytokine signalling-3 (SOCS-3) and p63. *MiR-203* starts to express in human foetal skin at week 17 and is most prominent in the suprabasal layers of the epidermis while p63 and SOCS-3 are preferentially expressed in the basal layer [96]. Differentiation-induced upregulation of *miR-203* expression is blocked by treatment of protein kinase C (PKC) inhibitor. The activator protein-1 (AP-1) proteins c-Jun and JunB regulate *miR-203* expression in keratinocytes.

Several studies are looking into the interaction between p63 and miRNAs in epidermis. In the absence of p63, *miR-34a* and *miR-34c* are increased in primary keratinocytes with concomitant G1-phase arrest, inhibition of cyclin D1 and cyclin-dependent kinase 4 (Cdk4). P63 inhibits the expression of *miR-34a* and *miR-34c* through directly binding to p53-consensus sites in regulatory regions of both genes [97]. In addition, the miR-17 family including *miR-17*, *miR-20b*, *miR-30a*, *miR-106a*, *miR-143*, and *miR-455-3p* are regulated by p63 [98].Multiple MAPKs, pRb and p21 are the direct target of these p63-regulated miRNAs. Inhibition of these miRNAs led to the defects in early keratinocyte differentiation. Thus, both p63-regulating miRNAs like *miR-203* and p63-regulated miRNAs constitute a feedback circuit in the regulation of epidermal differentiation.

As an inhibitory member of the ASPP (apoptosis stimulating protein of p53) family, iASPP comprise an autoregulatory feedback loop with p63 through microRNAs like *miR-574-3p* and *miR-720*. IASPP regulate epithelial integrity program by regulates the expression of genes essential for cell adhesion through affecting the expression of *miR-574-3p* and *miR-720* that directly target p63. Silencing of iASPP in keratinocytes by RNA interference promotes and accelerates a differentiation pathway [99]. In addition to p63, other epigenetic regulators like PcGs are also regulated by miRNAs to determine the proliferation and differentiation of epidermal stem cells. For instance, *miR-137* [100] and *miR-26a* [101] repress the expression of EZH2 which is appreciated as a crucial role in epidermal homeostasis and differentiation.

An additional *let-7* target *mLin41* (mouse homologue of lin-41) presents in epidermal stem cell niches. It is a potential contributor to the mutipotency regulatory circuit of *let-7* miRNA and its target gene *Lin-28*. MLin41 interferes with silencing of target mRNAs by *let-7* and cooperates with the mutipotency factor Lin-28 in suppressing let-7 activity, revealing a dual control mechanism regulating let-7 in epidermal stem cells [102].

When most studies concentrate on regulation of keratinocytes [103], some studies begin to check into other component of epidermis. One study shows that *miR-146a* is constitutively expressed at higher levels in human Langerhans cells (LC). It is induced by the transcription factor PU.1 in response to TGF-beta1, a key microenvironmental signal for epidermal LC differentiation. Besides, high constitutive *miR-146a* levels might render LCs less susceptible to inappropriate activation by commensal bacterial TLR2 triggers at body surfaces [104].

In addition to miRNAs, lncRNAs are postulated to play important roles in epidermal stem cells. Using transcriptome sequencing and tiling arrays to compare lncRNA expression in epidermal progenitor populations *versus* differentiating cells, researchers identified ANCR (anti-differentiation ncRNA) as an 855-base-pair lncRNA down-regulated during differentiation. Depletion of ANCR in epidermal stem cells leads to rapid induction of differentiation genes [105], indicating that the ANCR lncRNA is responsible for the maintenance of undifferentiated state of epidermal stem cells.

Functionally contrary to ANCR, a human lncRNA terminal differentiation-induced ncRNA (TINCR) has been characterized recently. TINCR-deficient epidermis lacks terminal differentiation ultrastructure, including keratohyalin granules and intact lamellar bodies. Through a post-transcriptional mechanism to ensure differentiation mRNAs expression, TINCR controls human epidermal differentiation. TINCR interacts with a range of differentiation mRNAs including FLG, LOR, ALOXE3, ALOX12B, ABCA12, CASP14 and ELOVL by TINCR-mRNA interaction occurring through a 25-nucleotide “TINCR box” motif. Direct binding of TINCR to the staufen1 (STAU1) protein is required for skin differentiation [106].

### 2.4. Epigenetic Regulations of Epidermal Stem Cells and Skin Disorders

As discussed above, dynamic epigenetic regulation has been proved crucial in epidermal stem cell differentiation and homeostasis maintaining. Recently, contribution of epigenetic changes and epidermal stem cells to skin disorders has attracted increasing attentions [107]. The development of cancer is a multistage long process resulting from the accumulation of genetic and epigenetic changes in the stem or progenitor cells [108]. Similarly, epidermal stem cells or melanocyte precursors undergo several epigenetic and genomic changes probably induced by UV (ultra violet) to eventually develop into melanoma cells. These changes provide stem cells with abilities to avoid apoptosis, replicate without limitation, sustain angiogenesis and invade and spread to other sites [108,109]. UV present in the sunlight exhibits deleterious effects on DNAs. Interestingly, methylated cytosines are much more susceptible to UV-induced cyclobutane pyrimidine dimers (CPDs) formation than unmethylated ones [110]. As a consequence of epigenetic and genetic changes, many signaling pathways important to the function of epidermal cells are dysregulated in melanoma cells. For example, murine double minute 2 (MDM2)-p53 pathway is important to maintain the number and function of epidermal stem cell [111]. The increase of p53 resulted from the deletion of MDM2 in the epidermis induced the loss of epidermal stem cell function and consequent aging phenotypes such as thin epidermis, retarded wound healing and a progressive loss of fur. During melanoma development, this pathway was inactivated by the loss of p53 function although the point mutation of p53 was relatively infrequent, indicating the potential implication of the epigenetic inactivation of p53 signaling. Indeed, MDM2-targeting *miR-18b* was epigenetically downregulated in human melanoma specimens and cell lines [112]. Stable overexpression of *miR-18b* greatly suppressed melanoma cell viability, cell migration and invasiveness, reversed epithelial-to-mesenchymal transition (EMT), and inhibited tumor growth *in vivo*. Meanwhile, some other well-known tumor suppressor genes such as PTEN and RARB are inactivated by DNA methylation in melanoma [113].

Defects in the epigenetic regulation of epidermal stem cells also contribute to the development of non-malignant skin diseases. Some characteristic cutaneous features present in patients with disruption of gene imprinting. These diseases include Beckwith–Wiedmann, Silver–Russell, Prader–Willi, McCune–Albright and Angelman syndromes, Albright’s hereditary osteodystrophy, progressive osseous heteroplasia, Von Hippel–Lindau syndrome, hypomelanosis of Ito and dermatopathia pigmentosa reticularis [114]. More common diseases include eczema and psoriasis may also have predominantly maternal or paternal modes of transmission. In addition to gene imprinting, other epigenetic changes are also relevant to the pathogenesis of skin diseases. For example, overexpression of *miR-31* contributes to skin inflammation in psoriasis lesions by regulating the production of inflammatory mediators and leukocyte chemotaxis to the skin [115]. Psoriasis can be characterized by a specific microRNA expression profile and *miR-31* is highly upregulated microRNAs in psoriasis skin by TGF-β (Tumor growth factor-β) which was predominant in psoriasis epidermis. It directly targets serine/threonine kinase 40 (STK40) and inactivates NF-κB signaling. Inhibition of *miR-31* activates NF-κB signaling and promotes the expression of many inflammatory cytokine or chemokines such as interleukin-1β (IL-1β), IL-8 and epithelial-derived neutrophil-activating peptide 78. In addition, the mammalian homolog of Grainyhead in Drosophila, Grainyhead-like 2 (GRHL2) regulates telomerase (hTERT) expression in epidermal cells. Interestingly, GRHL2 was also upregulated in chronic skin lesions such as psoriasis. Exogenous GRHL2 expression inhibited the recruitment of histone demethylase Jmjd3 to enhance the level of histone 3 Lys27 trimethylation enrichment at promoters and suppress the expression of epidermal differentiation complex genes (EDC) such as IVL, KRT1, FLG, LCEs, and SPRRs [116].

## 3. Summary

The dynamic epigenetic network including histone modifications, DNA modifications and non-coding RNAs regulate the stemness and plasticity of epidermal stem cells to maintain epidermal homeostasis. Epidermal stem cells are proposed as useful resource for stem cells used in either stem cell research or regenerative medicine. Although epigenetic networks in the regulation of epidermal stem cells remain to be clarified, modulation of epigenetic regulators has proven to have the potential for clinical application [85]. With the further understanding of epigenetic regulation of epidermal stem cells, more it will be of great significance to discover in the future how epidermal stem cells are epigenetically regulated in detail and what clinical implication does the epigenetic mechanism stand for.
